# Towards Integrated Physical Activity Profiling

**DOI:** 10.1371/journal.pone.0056427

**Published:** 2013-02-20

**Authors:** Dylan Thompson, Alan M. Batterham

**Affiliations:** 1 Department for Health, University of Bath, Bath, United Kingdom; 2 Health and Social Care Institute, Teesside University, Middlesbrough, United Kingdom; Ohio State University, United States of America

## Abstract

Recently, there has been some discussion of whether it is possible to score highly in one dimension of physical activity behaviour (e.g., moderate intensity exercise) whilst also scoring poorly in another (e.g., sedentary time). Interestingly, direct empirical observations to support these proposals are lacking. New technologies now enable the capture of physical activity thermogenesis on a minute-by-minute basis and over a sustained period. We used one of the best available technologies to explore whether individuals can score differently in various physiologically-important physical activity dimensions. We determined minute-by-minute physical activity energy expenditure over 7 days in 100 men aged 28±9 years. We used combined accelerometry and heart rate with branched equation modelling to estimate energy expenditure and extracted data for key physical activity outcomes and descriptors. Although some physical activity outcomes were tightly correlated, the attainment of one threshold for a given physical activity dimension did not automatically predict how well an individual scored in another dimension (with bivariate correlations ranging from 0.05 to 0.96). In one illustrative example of this heterogeneity, although 41 men showed a relatively low Physical Activity Level (total energy expenditure/resting energy expenditure ≤1.75), only 17% (n = 7) of these men showed consistently low physical activity across other dimensions (moderate intensity activity, vigorous intensity activity, and sedentary time). Thus, physical activity is highly heterogeneous and there is no single outcome measure that captures all the relevant information about a given individual. We propose that future studies need to capture (rather than ignore) the different physiologically-important dimensions of physical activity via generation of integrated, multidimensional physical activity ‘profiles’.

## Introduction

A low level of physical activity is a major public health problem that impacts upon most chronic diseases [1], [2]. In the past few years, there has been major progress in the technological assessment of physical activity energy expenditure. Several instruments estimate minute-by-minute energy expenditure (e.g., [3], [4]) and this will almost certainly become relatively standard in the future. We have previously shown that the application of such technologies with the capture of just one dimension (aspect) of physical activity behaviour leads to major discrepancies in terms of physical activity status [5]. Indeed, using the same raw data, approximately 90% of middle-aged men could be variably informed that they are both ‘active’ and ‘not sufficiently active’ [5]. Thus, it is extremely difficult to provide clear feedback to individuals in response to the important question “Am I doing enough of the right kind of physical activity for health?”.

We suspected that part of the discrepancy in individual classification was due to the highly individualised and unique signature profile associated with a given individual’s physical activity energy expenditure. Other authors have proposed that it is possible to score highly in one aspect of physical activity behaviour but low in another [6]–[8], although direct empirical observation of this phenomenon is lacking. The heterogeneity within physical activity behaviour becomes even more important when one takes into account the fact that various physical activity dimensions have independent biological and health benefits. For weight loss or maintenance, physical activity energy expenditure is the most important consideration and the nature (e.g., pattern and/or intensity) of the physical activity is not important [9]. However, in addition to thermogenesis, certain forms of physical activity generate profound independent health-related benefits. For example, short bouts of intense exercise produce significant metabolic gains without a major impact on total energy expenditure [10]–[12]. Bed rest studies show that even brief bouts of daily activity have the capacity to prevent the unravelling of metabolic homeostasis to sustained inactivity [13]. Epidemiological studies show that sedentary time and breaks in sedentary time may be independently important [14]–[16].

Thus, whilst new technologies create opportunities for the provision of personalised information regarding physical activity status, we envisage that there will be a need to confront the heterogeneous nature of physical activity in order to provide individuals with meaningful and personalised information regarding the appropriateness of their behaviour. Based on our earlier observations, we propose that some individuals will score highly in one physical activity dimension (e.g., time engaged in moderate intensity physical activity in bouts of 10 minutes) but low in another (e.g., total physical activity energy expenditure). In the present study, we set out to explore the extent of this potential heterogeneity in physical activity according to physiologically-important physical activity descriptors and dimensions.

## Methods

### Ethics Statement

The Bath Research Ethics Committee, part of the National Research Ethics Service, approved this research and all participants provided written informed consent prior to participation.

### Participants

One hundred men were recruited from the local community via posters and advertisements. Volunteers were healthy asymptomatic non-smokers who were not taking medication and had a Body Mass Index (BMI) ≤35 kg/m^2^. Mean (SD) age, height, body mass and BMI were 28 (9) years, 1.77 (0.08) m, 77.1 (13.2) kg and 24.5 (3.2) kg/m^2^.

### Experimental Design

We set out to dissect physical activity energy expenditure according to common physiologically-important physical activity descriptors that have been associated with positive/negative health.

### Assessment of Physical Activity Energy Expenditure

Minute-by-minute physical activity energy expenditure was estimated over a representative seven-day period using synchronized accelerometry and heart rate with branched equation-modelling (Actiheart, Cambridge Neurotechnology Ltd., Cambridge, UK) as previously described [3], [17], [18]. Data were recorded continuously throughout this period (i.e., day and night). Participants were instructed to remove the physical activity monitor only to change the ECG electrodes. A recording for a given individual was only accepted if heart rate data was available for at least 23 hours on each day of recording.

### Moderate to Vigorous Intensity Physical Activity

As described in detail previously [5], we used in-house software to determine the amount of time (minutes) engaged in physical activity above and below specific moderate and vigorous intensity thresholds (e.g., 3 Metabolic Equivalents or METs).

### Highly Vigorous Intensity Physical Activity

Recently, strong evidence has emerged that short bouts of high-intensity intermittent exercise have profound metabolic and health benefits that are similar to much longer bouts of prolonged exercise [10]–[12], [19]–[21]. For example, as little as 10×1 min bouts of exercise at ∼80–90% maximal oxygen uptake performed three times per week has enormous benefits [21]; and very high intensity exercise of less than one minute three times per week also has potent effects on metabolic control [12]. These forms of physical activity would not meet any physical activity recommendation, would not have a major impact on physical activity energy expenditure or total energy expenditure and would not impact upon total sedentary time. As far as we are aware, no one has attempted to capture or define these activities in free-living conditions. For the purpose of the present comparison, we determined total time engaged in physical activity greater or equal to 10.2 METs; defined as ‘very hard’ and equivalent to approximately 85% maximal oxygen uptake in an average person [22], [23].

### Sedentary Time

Recent studies and commentaries highlight the importance of sedentary behaviour for health [6], [14]–[16]. Sedentary behaviour is not just the absence of physical activity (e.g., the absence of activity greater than 3 METs) and is defined as activities requiring very low energy expenditure between 1 and 1.5 METs [7]. The recent guidelines from the Department of Health in the UK include the statement that people should “…aim to minimise the time they spend being sedentary each day” [24]. This report highlights the variability in the literature and methods for the assessment of sedentary time which precludes the development of a clear recommendation [24]. In the present study, in the absence of definitive information, we use two variants for comparison (i) spending greater than 60% of the waking day engaged in activities between 1–1.5 METs (reported as average sedentary time in some studies [15], [16]) and (ii) spending greater than 6 h a day engaged in activities between 1–1.5 METs (this amount of sedentary time has been reported to be strongly associated with risk of obesity and type 2 diabetes [25] and weight gain [26]). Without other contextual information, the separation of sedentary time from sleeping time using minute-by-minute estimates of energy expenditure is somewhat imprecise. In a subgroup (n = 14), we estimated daily waking time based on visual inspection of daily physical activity records. We found that estimated waking time was 15.9±0.5 h (15.3 to 16.4 h) and, given the imprecision of this estimate and the relative consistency between individuals, we subsequently assumed an 8 hour period of sleep for all participants and subtracted this from total time engaged in activity between 1 and 1.5 METs.

### Physical Activity Recommendations

We examined the ACSM/CDC and US Surgeon General recommendations that were used widely for over ten years [22], [27], the revised recommendations from ACSM/AHA published in 2007 [28], early recommendations from the UK Chief Medical Officer and Department of Health (DoH) published in 2004 [29], current recommendations from USDHHS/CDC [30], [31] that have also been adopted in the UK by the Department of Health [32], the current recommendations from the US Institute of Medicine [33] and recommendations from the World Health Organisation (WHO) [34]. Some recommendations are expressed using multiple outcomes (e.g., time engaged in moderate intensity physical activity *vs.* energy expenditure) or there are multiple subtly-different interpretations of the same recommendation. Where practical, we include some of these different permutations. Note that earlier recommendations [22], [23] include age-specific thresholds for moderate intensity physical activity (4.8 METs) and vigorous intensity physical activity (7.2 METs) that differ from the more ubiquitous 3 and 6 MET thresholds used in many other recommendations. We describe interpretation and analysis of these recommendations in detail elsewhere [5].

### Data Analysis

Data for the various physiologically-important physical activity descriptors and recommendations were collated and depicted at an individual level. Our target sample size was based closely on our previous work (37) and was considered adequate to provide sufficient information on the multidimensional physical activity profiles to inform future work. We provide descriptive statistics for various physical activity outcomes and used Pearson’s correlations to examine the relationships between each metric.

## Results

### Physical Activity

Mean energy expended through daily physical activity was 1172 kcal (range 464–2559 kcal) with a mean PAL of 1.85 (range 1.37–2.45). The mean time engaged in moderate intensity activity greater than 3 METs per day was 143 minutes (range 21–327 minutes), which was reduced to 64 minutes per day if we only count activity accumulated in bouts of 10 minutes or more (range 0–160 minutes). The mean time engaged in vigorous intensity activity greater than 6 METs per day was 23 minutes (range 0–71 minutes), or 14 minutes per day for vigorous intensity activity accumulated in bouts of 10 minutes or more (range 0–52 minutes). The mean time engaged in highly vigorous intensity activity >10.2 METs was 6 minutes (range 0–44 minutes). The mean percentage of the day spent sedentary was 52% (range 15–82%).

### Relationships between Different Physical Activity Dimensions

**Figure 1:**
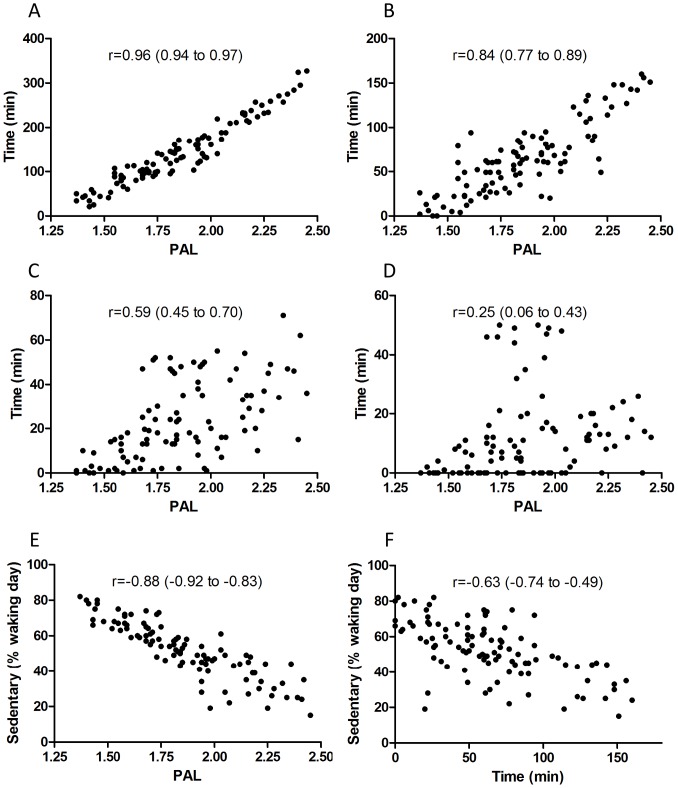
Example relationships between various physical activity dimensions or attributes. **A**, PAL *versus* daily time engaged in physical activity >3 METs accumulated on a minute-to-minute basis; **B**, PAL *versus* daily time engaged in physical activity >3 METs accumulated in bouts of at least 10 min; **C**, PAL *versus* daily time engaged in physical activity >6 METs accumulated in bouts of at least 10 min; **D**, PAL *versus* daily time engaged in physical activity >7.2 METs accumulated in bouts of at least 10 min; **E**, PAL *versus* daily time engaged in sedentary activities as a proportion of the waking day (i.e., below 1.5 METs accumulated on a minute-to-minute basis); **F**, daily time engaged in physical activity >3 METs accumulated in bouts of at least 10 min *versus* daily time engaged in sedentary activities as a proportion of the waking day (i.e., below 1.5 METs accumulated on a minute-to-minute basis). Pearson correlations (95% confidence interval) are reported.

Some dimensions of physical activity were very tightly correlated such as PAL and time engaged in physical activity above 3 METs accumulated on a minute-to-minute basis (Table 1 and Figure 1A). For most other physical activity dimensions, the strength of these relationships was diminished and more variable (Table 1). Visual inspection of these relationships (e.g., Figure 1B) show that some individuals had a very high PAL (∼2.00) but spent little time engaged in physical activity above 3 METs in bouts of at least 10 min; and there are other individuals who accumulate considerable amounts of time engaged in physical activity above 3 METs in bouts of at least 10 min but where this is insufficient to increase PAL to ‘active’ levels of greater than 1.75 (Figure 1B). These discrepancies are even more pronounced for vigorous intensity activity and sedentary time (Table 1 and Figure 1C–F).

**Table 1:** A correlation matrix showing various commonly-used physiologically-important physical activity descriptors (n = 100).

	Mod/d(>3 METs)	Mod/wk(>3 METs^10^)	Vig/d(>6 METs)	Vig/d(>6 METs^10^)	Vig/d(>7.2 METs^10^)	Vig/d(>10.2 METs)	MET^.^min/wk	% sedentary
PAL	0.96	0.84	0.59	0.32	0.25	0.41	0.80	−0.88
Mod/d (>3 METs)		0.88	0.47	0.16	0.31	0.39	0.77	−0.83
Mod/wk (>3 METs^10^)			0.67	0.29	0.29	0.39	0.95	−0.63
Vig/d (>6 METs)				0.84	0.78	0.63	0.84	−0.34
Vig/d (>6 METs^10^)					0.98	0.64	0.59	−0.10
Vig/d (>7.2 METs^10^)						0.60	0.53	−0.05
Vig/d (>10.2 METs)							0.58	−0.11
MET min/wk								−0.52

PAL: Weekly Physical Activity Level (total energy expenditure/resting energy expenditure), Mod/d (≥3 METs): Daily moderate intensity activity above 3 METs assessed on a minute-by-minute basis, Mod/wk (≥3 METs^10^): Weekly moderate intensity activity above 3 METs in bouts of at least 10 minutes, Vig/d (≥6 METs): Daily vigorous intensity activity above 6 METs assessed on a minute-by-minute basis, Vig/d (≥6 METs^10^): Daily vigorous intensity activity above 6 METs in bouts of at least 10 minutes Vig/d (≥7.2 METs^10^): Daily vigorous intensity activity above 7.2 METs in bouts of at least 10 minutes, Vig/d (≥10.2 METs): Daily vigorous intensity activity above 10.2 METs assessed on a minute-by-minute basis, MET min/wk: total MET min/wk for activity ≥3 METs in bouts of 10 min or more, % sedentary: percentage of the day spent sedentary after adjusting for sleep (≤1.5 METs).

### Individual Attainment of Defined Physical Activity Attributes/Thresholds

**Figure 2:**
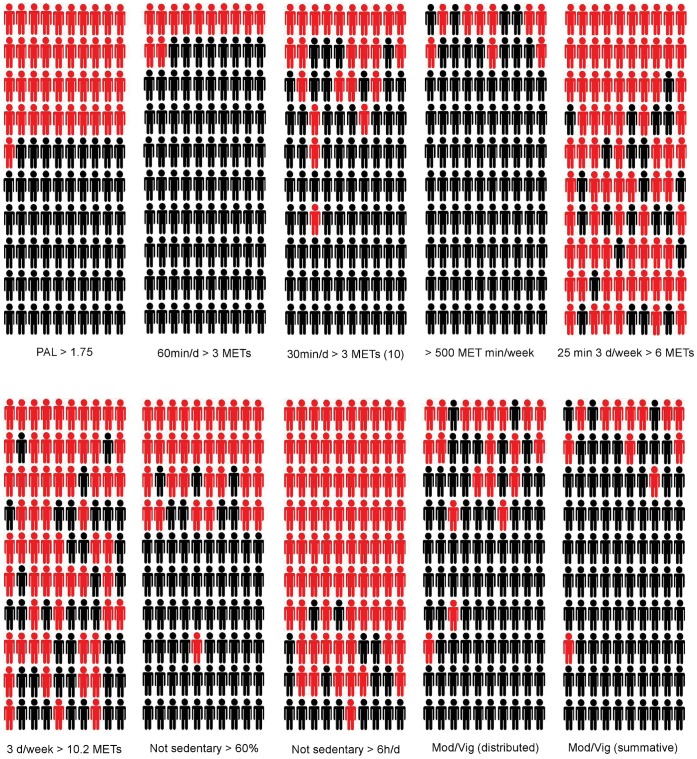
Heterogeneity in physical activity status according to dimension or characteristic. The data for 100 men is shown in rank order for PAL with each individual retaining their relative position for all other dimensions/characteristics. Red indicates below and black indicates above the defined threshold for each attribute/characteristic.

At an individual level, the heterogeneity in physical activity influences the classification of individuals according to commonly-used physical activity descriptors (See Figure 2 and Table 2). Broadly, the most and least active men defined by PAL (e.g., the highest 10 and lowest 10) tended to exceed or fail to exceed each of the given thresholds for other recommendations/guidelines, respectively (Figure 2 and Table 2). However, even at these extremes, this was not entirely consistent with, for example, four of the 10 least active men according to PAL exceeding the threshold of 500 MET/min per week (Figure 2 and Table 2). Of the 41 men who showed a relatively low Physical Activity Level (≤1.75), only 17% (n = 7) showed consistently low physical activity across all other dimensions (Figure 2 and Table 2). Some men achieve substantive amounts of vigorous intensity physical activity and yet this is insufficient to increase their overall PAL. Clearly, an individual scoring highly in one particular physical activity dimension will not necessarily score well in another dimension.

**Table 2:** Definitions of the key physical activity dimensions included in Figure 2.

Physical Activity Dimensionor Characteristic	Definition
PAL	A Physical Activity Level (PAL) >1.75
60 min/d >3 METs	60 min of moderate intensity activity (>3 METs) on average per day (accumulated in 1 min epochs)
30 min/d >3 METs (10)	30 min of moderate intensity activity (>3 METs) on average per day (accumulated in bouts of at least 10 min)
>500 MET min/week	500 MET min/week ≥3 METs in bouts of 10 min or more
25 min 3d/week >6 METs	25 min of vigorous intensity physical activity (>6 METs) on at least 3 days per week in bouts of at least 10 min
3 d/week >10.2 METs	1 min of highly vigorous intensity physical activity (>10.2 METs) on at least 3 days per week
Not sedentary >60%	60% of the waking day (16 h) on average spent below 1.5 METs (accumulated in 1 min epochs)
Not sedentary >6 h/d	6 h of the waking day (16 h) on average spent below 1.5 METs (accumulated in 1 min epochs)
Mod/vig (distributed)	30 min of moderate intensity activity on at least 5 days in bouts of 10 min or 20 min of vigorous intensity activity on at least 3 days in bouts of 10 min; or a combination
Mod/vig (summative)	150 min of moderate intensity physical activity (3–6 METs) or 75 min of vigorous intensity activity (≥ METs) per week in bouts of at least 10 min; or a proportional combination of moderate and vigorous intensity activity to meet a combined target.

### Physical Activity Recommendations

The proportion of men meeting the various physical activity recommendations in the present study ranged from 18% to 91% (Figure S1). The median proportion (interquartile range) of men defined as sufficiently active across all recommendations was 73% (41% to 88%).

## Discussion

The results of the present study confirm the highly heterogeneous and multi-dimensional nature of physical activity. It appears unlikely that there is a single outcome measure that captures all the relevant information about physical activity since a given individual can show high physical activity when using one particular metric but low physical activity (or high sedentary time) when using a different outcome or descriptor.

### Physical Activity is Inherently Heterogeneous

The capture of only one physical activity descriptor or dimension will inevitably omit other aspects of the behaviour that could be equally important from a physiological perspective. As shown in Figure 1, some men achieve very high physical activity energy expenditure values (i.e., PAL) via considerable activity below 3 METs; presumably in the form of Non-Exercise Activity Thermogenesis [9]. Whilst this type of activity might not meet classical and current physical activity recommendations, previous research indicates that it would offer powerful and distinct health benefits [8], [9]. Our results also show that some individuals participate in substantial amounts of vigorous intensity physical activity (presumably structured exercise) but otherwise have a relatively low overall physical activity energy expenditure. Again, given the powerful effects of vigorous intensity physical activity on various health outcomes [23], [28], this physical activity profile would presumably be associated with considerable net health benefit. Furthermore, it is clear that whilst some men spend large amounts of the day engaged in sedentary behaviour, they still manage to achieve very high physical activity energy expenditure (presumably due to participation in relatively short episodes of high intensity exercise); and, again, this would probably be sufficient to confer health benefits [23]. In summary, these results show that it is possible to be highly active according to one metric but, at the same time, labelled as insufficiently active or sedentary according to another. We provide individual examples in the supplementary information (Figure S2). Clearly, physical activity is not a dichotomous behaviour and, further, it seems highly likely that physical activity cannot even be measured on a single continuum. Given the likely variability in the physical activity of our hunter-gatherer ancestors [35], multiple wholly-different physical activity patterns and profiles would be entirely normal from an evolutionary perspective.

### Uni-dimensional Physical Activity Overlooks Potentially Important Diversity and Heterogeneity

Whilst the assessment of one physical activity dimension will provide some information about the totality of the behaviour, the strength of the relationship will inevitably be confounded by this heterogeneity. As a result, many individuals will be miscategorised and inappropriately labelled. This has implications for epidemiologists. For example, individuals who score poorly in terms of PAL should not necessarily be treated as ‘less active’ if they score highly for participation in vigorous intensity exercise (since this could be physiologically important). Equally, these observations have implications for scientists planning intervention studies. For example, two ‘inactive’ people identified according to low participation in physical activity above 3 METs in bouts of 10 min could have highly divergent physical activity energy expenditure (i.e., PAL); and thus might not be expected to respond in the same way to a given intervention. Whether this plays a part in explaining some of the individual variability in physiological and health-related outcomes that has been documented in response to training studies is an open question [36].

### Multi-dimensional Physical Activity Profiling Provides Novel Opportunities

Technological progress means that it is now possible to capture various physical activity dimensions during free-living conditions and thus we are in the position to improve the resolution of feedback for individuals. Clearly, this will require studies that tease out which dimensions of physical activity are biologically-linked to health-related outcomes in various populations. It will also be important to ensure that the various dimensions capture something unique. For example, based on our current data, PAL and time engaged in physical activity above 3 METs on a minute-by-minute basis (i.e., not in 10 minute bouts) were very closely related. Moreover, our tentative receiver operating characteristic (ROC) curve analysis (not shown) indicates that if a given individual spends more than 116 minutes engaged in physical activity above 3 METs then PAL will nearly-always exceed 1.75; with a sensitivity and specificity of 95%. Based on these initial observations, we might not need to include both of these physical activity outcomes or dimensions in a physical activity profile. Ultimately, the goal might be to use various physical activity attributes (inputs) to derive a more complete picture in a similar way to the criteria used to define the metabolic syndrome (e.g., if 3 out of 5 dimensions are ‘negative’ or ‘low’ then this indicates increased risk of chronic disease). Clearly, the present study is only a first step and much more work will be required to develop a truly meaningful profile. Future studies need to explore the relationships between the various aspects of a potential profile and health-related outcomes such as risk factors for cardiovascular disease. It will be important to consider whether some aspects of the profile should be weighted differently and that the effects of each component are truly additive. It will also be important to determine whether the presence of a pre-defined number of low scores in specific dimensions can be used to determine an analogue of the metabolic syndrome (e.g., Physical Inactivity Syndrome). The present study cannot answer these questions but PAL, moderate intensity activity in bouts of 10 min, vigorous intensity activity above 6 METs in bouts of 10 min, highly vigorous intensity activity (similar to high-intensity interval training) and sedentary time all have positive effects on health [1], [6], [10]–[12], [14]–[16], [19], [24], [26], [31], [32], [34] and, based on the results in the current analysis, it is quite feasible that a given individual will score highly in one or more of these dimensions and low in another. Thus, we propose that we have the starting point for an integrated physical activity profile that will more accurately capture an individual’s risk of chronic disease. The next step will be to design appropriate epidemiological studies to tease out the important categories, to identify whether any weighting is required, and to determine whether a specific combination of scores is more predictive of health outcomes such as risk factors for cardiovascular disease and type 2 diabetes than single descriptors alone.

### Homogeneous Uni-dimensional Physical Activity Undermines the Quality of Personalised Feedback

**Figure 3:**
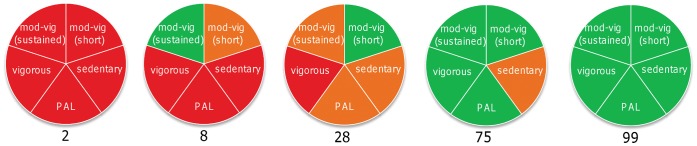
A possible example of how physical activity profiles might look in the future. Each profile captures five different dimensions for five of the participants in the present study (participants 2, 8, 28, 75 and 99 based on their relative position depicted in Figure 2). The five dimensions or characteristics are: (1) a Physical Activity Level ≥1.75, (2) participation in at least 150 minutes of moderate-vigorous intensity activity (3–6 METs) or 75 minutes of vigorous intensity activity (>6 METs) per week in bouts of at least 10 minutes; or a proportional combination of moderate and vigorous intensity activity to meet a combined target (3) participation in at least 60 minutes of moderate intensity activity (>3 METs) on average per day accumulated on a minute-to-minute basis (4) participation in 25 minutes of vigorous intensity activity in bouts of at least 10 minutes on at least 3 different days of the week and (5) participation of less than 60% of the waking day per week spent engaged in activities below 1.5 METs accumulated on a minute-to-minute basis). In this simple example iteration, we have used green/red to indicate the clear achievement/failure to achieve each threshold; with yellow indicating that values were within approximately 20% of the target value.

The highly-individualised and heterogeneous signature profile for physical activity has implications for personalised feedback to individuals. The most obvious consequence is that if people are provided with just one physical activity descriptor or dimension, then they will be potentially misinformed about the appropriateness of their behaviour. Equally, clinicians could inadvertently form an inappropriate conclusion about a given patient. As discussed above, whilst we are not yet in a position to advocate definitive dimensions, we have explored one possible very simple formulation in Figure 3. At an individual level, the increased sensitivity obtained from a profile should be balanced against the danger that people find the additional information either confusing or unhelpful. In this example, we have borrowed from the ‘traffic light’ system used for labelling foods in the UK and elsewhere around the world to generate a simple colour-coded physical activity profile that captures multiple dimensions of physical activity behaviour. This schematic shows five different individuals from the current sample of young men. If these individuals were provided with feedback on just one aspect or dimension of their behaviour, we may form a very different conclusion depending on the metric that is being used. Other investigators have eloquently described how it is possible for an individual to be simultaneously classed as both sedentary and active [6], [7]. Notably, in this particular sample of young men and using this simple formulation, seven individuals had an entirely ‘red’ profile and four had entirely ‘green’ profiles. Thus, the vast majority undertook some (variable) physical activity in one or more dimensions. This simple visual representation demonstrates that it is feasible to provide feedback on multi-dimensional physical activity in a straightforward and readily-understandable manner. Of course, there will be other options and the use of colour-coding in this example (without a sense of magnitude) faces some of the same problems as the traffic light system for food/diet. Furthermore, whilst such a simple profile might work well for the provision of personalised feedback to individuals, it would be much more useful if we could combine this with a summative risk score derived from an assessment across the various dimensions (although, as discussed above, this will only be possible once the necessary studies have been completed).

### Technological Innovation will Enable Physical Activity Profile Selection and Calibration

It is noteworthy that, at first glance, the young men recruited in the present study appear extremely active when viewed in the context of existing physical activity guidelines. However, it is important to highlight that we have objectively monitored weekly physical activity for almost every minute of every day whereas, in the past, most investigations only captured certain elements of physical activity behaviour such as walking or leisure time physical activity. Thus, recommendations that have been derived from relative ‘snapshots’ of physical activity will probably need recalibrating following the introduction of new techniques that capture and provide feedback for the totality of the behaviour. This has been discussed previously [5], [37], [38]. Importantly, when these results are compared to other studies that have used similar techniques, the young men recruited in the present study are only modestly more active than middle-aged men and women [38], [39]; which might be anticipated given their younger age. Based on the preceding discussion, we may need to revisit and recalibrate certain thresholds that are used for some physical activity dimensions (e.g., time engaged in moderate intensity activity) in order to build a satisfactory physical activity profile in the future.

Of course, based on the results of the present study alone, we do not know whether this situation is better or worse in other populations or in groups that are generally more or less active. In addition, we have not included physical activity between 1.5 to 3 METs in the present study and this could be physiologically important. We have also excluded other aspects to physical activity behaviour that might only contribute a small amount to physical activity energy expenditure but that could, nonetheless, lead to very specific and beneficial adaptations (e.g., resistance exercise). Finally, water-based activities such as swimming would not be accurately quantified using the current technologies and we have not taken this into account in the current study – although any notable bout of prolonged swimming greater than one hour would have resulted in exclusion based on the lack of heart rate data.

### Conclusion

To date, physical activity has typically been captured in uni-dimensional terms (e.g., time engaged in moderate intensity activity). In the present study, we confirm that a given individual can score highly in one physical activity dimension but poorly in another. With the advent of new technologies, a physical activity assessment now generates thousands of data points which can be dissected and analysed in dozens of different ways. Rather than reducing this to just one single outcome measure or descriptor, we propose that we need novel approaches to capture (rather than ignore) the different physiologically-important dimensions of physical activity via generation of integrated, multidimensional physical activity ‘profiles’.

## Supporting Information

Figure S1 **The proportion of men in this sample who either met or failed to meet each of the 14 recommendations included in the present study (n = 100).**
(PPT)

Figure S2 **Example daily energy expenditure for five different individuals illustrating some of the heterogeneity inherent in key physical activity outcomes.** Individuals A and D have a similar PAL but have clearly achieved this in very different ways – and the capture of PAL alone would not illustrate the difference in other dimensions. Individual B engages in twice as much moderate to vigorous intensity activity as Individual D and, yet, has a lower overall physical activity energy expenditure (i.e., PAL). Individual D spends most of the day engaged in sedentary activity – but one single bout of vigorous intensity activity is sufficient to have a major impact on PAL. Individual E shows the highest moderate intensity activity –but otherwise scores relatively poorly in other dimensions (etc). Time represents minutes from midnight. Each summative outcome is for the specific day that is depicted. PAL: Physical Activity Level, METs: Metabolic Equivalents, Not Sedentary: Percentage of waking day spent below 1.5 METs, >3 and >6 METs^10^: only activity above these thresholds in bouts of at least 10 minutes is counted. The horizontal dotted lines indicate 3 and 6 MET intensity thresholds for each individual.
(DOCX)
