# Supplementary material for: Towards Integrated Physical Activity Profiling
Source: PLoS One. 2013 Feb 20;8(2):e56427. doi: 10.1371/journal.pone.0056427 (PMC3577906; doi:10.1371/journal.pone.0056427)
Supplement: Figure S1 — The proportion of men in this sample who either met or failed to meet each of the 14 recommendations included in the present study (n = 100). (PPT) [file pone.0056427.s001.ppt]

## Slide 1
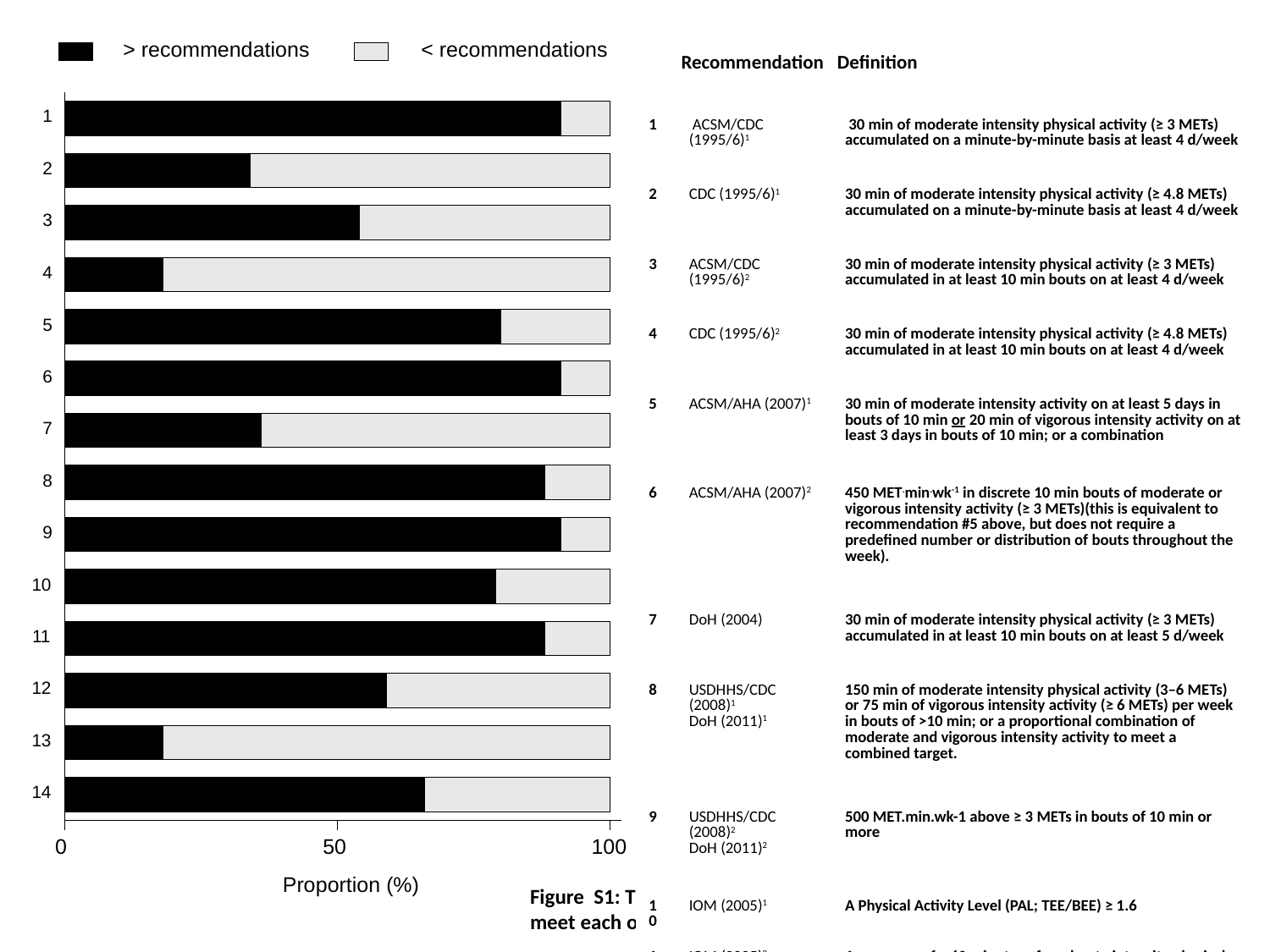

| | Recommendation | Definition |
| --- | --- | --- |
| 1 | ACSM/CDC (1995/6)1 | 30 min of moderate intensity physical activity (≥ 3 METs) accumulated on a minute-by-minute basis at least 4 d/week |
| 2 | CDC (1995/6)1 | 30 min of moderate intensity physical activity (≥ 4.8 METs) accumulated on a minute-by-minute basis at least 4 d/week |
| 3 | ACSM/CDC (1995/6)2 | 30 min of moderate intensity physical activity (≥ 3 METs) accumulated in at least 10 min bouts on at least 4 d/week |
| 4 | CDC (1995/6)2 | 30 min of moderate intensity physical activity (≥ 4.8 METs) accumulated in at least 10 min bouts on at least 4 d/week |
| 5 | ACSM/AHA (2007)1 | 30 min of moderate intensity activity on at least 5 days in bouts of 10 min or 20 min of vigorous intensity activity on at least 3 days in bouts of 10 min; or a combination |
| 6 | ACSM/AHA (2007)2 | 450 MET.min.wk-1 in discrete 10 min bouts of moderate or vigorous intensity activity (≥ 3 METs)(this is equivalent to recommendation #5 above, but does not require a predefined number or distribution of bouts throughout the week). |
| 7 | DoH (2004) | 30 min of moderate intensity physical activity (≥ 3 METs) accumulated in at least 10 min bouts on at least 5 d/week |
| 8 | USDHHS/CDC (2008)1 DoH (2011)1 | 150 min of moderate intensity physical activity (3–6 METs) or 75 min of vigorous intensity activity (≥ 6 METs) per week in bouts of >10 min; or a proportional combination of moderate and vigorous intensity activity to meet a combined target. |
| 9 | USDHHS/CDC (2008)2 DoH (2011)2 | 500 MET.min.wk-1 above ≥ 3 METs in bouts of 10 min or more |
| 10 | IOM (2005)1 | A Physical Activity Level (PAL; TEE/BEE) ≥ 1.6 |
| 11 | IOM (2005)2 | An average of ≥ 60 minutes of moderate intensity physical activity (≥3 METs) per day |
| 12 | WHO | A Physical Activity Level (PAL; TEE/BEE) ≥ 1.75 |
| 13 | Not Sedentary | Less than 6 hours of the waking day spent engaged in sedentary activities (i.e., activities ≤ 1.5 METs; assessed on a minute-by-minute basis) |
| 14 | Not sedentary | Less than 60% of the waking day spent engaged in sedentary activities (i.e., activities ≤ 1.5 METs; assessed on a minute-by-minute basis) |
> recommendations
< recommendations
1
2
3
4
5
6
7
8
9
10
11
12
13
14
0
50
100
 Proportion (%)
Figure S1: The proportion of men in this sample who either met or failed to meet each of the 14 recommendations included in the present study (n = 100).
