# Supplementary material for: Towards Integrated Physical Activity Profiling
Source: PLoS One. 2013 Feb 20;8(2):e56427. doi: 10.1371/journal.pone.0056427 (PMC3577906; doi:10.1371/journal.pone.0056427)
Supplement: Figure S2 — Example daily energy expenditure for five different individuals illustrating some of the heterogeneity inherent in key physical activity outcomes. Individuals A and D have a similar PAL but have clearly achieved this in very different ways – and the capture of PAL alone would not illustrate the difference in other dimensions. Individual B engages in twice as much moderate to vigorous intensity activity as Individual D and, yet, has a lower overall physical activity energy expenditure (i.e., PAL). Individual D spends most of the day engaged in sedentary activity – but one single bout of vigorous intensity activity is sufficient to have a major impact on PAL. Individual E shows the highest moderate intensity activity –but otherwise scores relatively poorly in other dimensions (etc). Time represents minutes from midnight. Each summative outcome is for the specific day that is depicted. PAL: Physical Activity Level, METs: Metabolic Equivalents, Not Sedentary: Percentage of waking day spent below 1.5 METs, >3 and >6 METs10: only activity above these thresholds in bouts of at least 10 minutes is counted. The horizontal dotted lines indicate 3 and 6 MET intensity thresholds for each individual. (DOCX) [file pone.0056427.s002.docx]

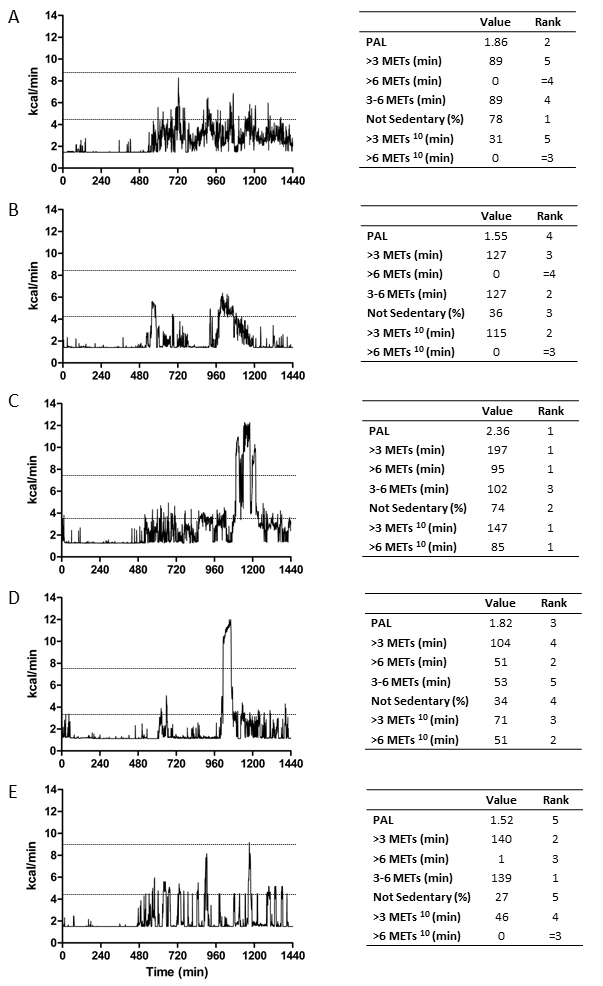


**Figure S2:** Example daily energy expenditure for five different individuals illustrating some of the heterogeneity inherent in key physical activity outcomes. Individuals A and D have a similar PAL but have clearly achieved this in very different ways – and the capture of PAL alone would not illustrate the difference in other dimensions. Individual B engages in twice as much moderate to vigorous intensity activity as Individual D and, yet, has a lower overall physical activity energy expenditure (i.e., PAL). Individual D spends most of the day engaged in sedentary activity – but one single bout of vigorous intensity activity is sufficient to have a major impact on PAL. Individual E shows the highest moderate intensity activity –but otherwise scores relatively poorly in other dimensions (etc). Time represents minutes from midnight. Each summative outcome is for the specific day that is depicted. PAL: Physical Activity Level, METs: Metabolic Equivalents, Not Sedentary: Percentage of waking day spent below 1.5 METs, >3 and >6 METs^10^: only activity above these thresholds in bouts of at least 10 minutes is counted. The horizontal dotted lines indicate 3 and 6 MET intensity thresholds for each individual.
